# Use of health services in people with multiple sclerosis with and without depressive symptoms: a two-year prospective study

**DOI:** 10.1186/1472-6963-13-365

**Published:** 2013-09-28

**Authors:** Charlotte Ytterberg, Sanna Lundqvist, Sverker Johansson

**Affiliations:** 1 Department of Neurobiology, Care Sciences and Society, Karolinska Institutet, Huddinge, Sweden; 2 Department of Clinical Neuroscience, Karolinska Institutet, Stockholm, Sweden; 3 Department of Physical Therapy, Karolinska University Hospital, Stockholm, Sweden; 4 Division of neurology R54, Karolinska University Hospital, Huddinge, Stockholm S-141 86, Sweden

**Keywords:** Multiple sclerosis, Depressive symptoms, Longitudinal studies, Prospective studies, Health services, Observational

## Abstract

**Background:**

To organize tailored healthcare for people with multiple sclerosis (MS), knowledge about patterns in the use of healthcare among subgroups, such as those with depressive symptoms, is essential. Thus, the purpose of this study was to explore and compare the use of health services in people with MS and depressive symptoms, and without depressive symptoms over a period of 30 months.

**Methods:**

Data on the use of health services by 71 people with MS and depressive symptoms, and 102 with no depressive symptoms were collected from a computerised register and by interview, then categorized with regard to disease severity (Expanded Disability Status Scale).

**Results:**

People with EDSS mild and depressive symptoms used more outpatient and inpatient care compared to those with no depressive symptoms. Furthermore, they received more unsalaried informal care as well as intense rehabilitation periods.

**Conclusions:**

The issues underlying the differences in the use of healthcare need to be explored further, as well as the plausible implications for the organization of healthcare services for people with MS and depressive symptoms. Furthermore, the life situations of caregivers of people with MS and depressive symptoms should be considered, and appropriate interventions supplied in order to diminish caregiver burden.

## Background

Studies on the use of healthcare among people with multiple sclerosis (MS) show that large proportions use hospital care and primary care in parallel with many different departments and services involved [1]. In addition, the use of healthcare may vary in different subgroups of people with MS, as seen with regard to fatigue [2], and depressive symptoms [3], for example, although results are conflicting [4].

Several studies show that the prevalence of depression among people with MS is higher compared to the general population [5], varying between 26% and 42% [5-7], as well as when compared to people with other chronic conditions [5]. There is evidence that depressive symptoms in people with MS are strongly associated with worse self-reported functioning and low health-related quality of life [8,9]. Furthermore, associations between depressive symptoms and fatigue have also been reported [10,11], as well as associations with impaired cognition, although results are inconsistent [7,12]. People with MS and depressive symptoms have a lower coping capacity [7] use less-effective coping strategies, (e.g., avoidance) [13], and also perceive less social support from their families and friends [14]. It has also been found that they have more lost days from work [15]. Depressive symptoms can negatively impact the progression of MS, since depressed people with MS are less likely to adhere to immunomodulatory treatment [16]. Depressive symptoms in people with MS have also been found to be associated with death by suicide [17].

Recommendations of treatment for depression in MS include pharmacologic or psychotherapeutic approaches, or preferably a combination of both [18]. Nevertheless, depressive symptoms have been found to be undertreated [19,20], and despite a high level of general satisfaction with healthcare in people with MS, many experience a lack of psychosocial support [21]. Furthermore, the use of healthcare has been shown to vary in different subgroups of people with MS [2], but it is not known whether the use varies also among people with MS with or without depressive symptoms. Therefore, to plan for and organize tailored healthcare for people with MS, it is important to have knowledge about patterns in the use of healthcare among different subgroups, such as those who also have depressive symptoms. Thus, the aim of this study was to explore and compare the use of health services in people who have MS and depressive symptoms, and without depressive symptoms over a period of 30 months.

## Methods

### Participants and procedures

Data were collected in the context of a prospective, observational study of functioning, perceived health and the use of healthcare among people with MS. All people with MS who were scheduled for an outpatient visit with one of two senior neurologists at the MS Centre of the Department of Neurology at Karolinska University Hospital, Huddinge, in Stockholm, Sweden during the period from February 1, 2002 to June 12, 2002, were eligible for inclusion and 219 people with MS were included in the study after securing informed consent. Follow-ups were performed every six months for two years, with 200 people completing the full study. During inclusion, and then at 6, 12, 18 and 24 months, the people with MS met an investigator, one of five research physiotherapists trained to perform the tests according to a standardized procedure, primarily the same investigator, and at the same time of day on all occasions. Detailed descriptions of demographic and disease-related data in the sample have been reported elsewhere [22]. The study was approved by the ethical review board of Karolinska Institutet in Stockholm, Sweden.

### Data collection

The neurologist responsible assessed disease severity using the Expanded Disability Status Scale (EDSS) [23]; scores were categorized as EDSS mild (EDSS 0–3.5), EDSS moderate (EDSS 4.0–5.5) or EDSS severe (EDSS 6.0–9.5) and determined the disease course (relapsing remitting, secondary progressive, primary progressive). The remaining data were collected by the research physiotherapists. Demographic- and disease-related data were collected both by means of interviews and from the medical records.

The Beck Depression Inventory (BDI) [24] was employed at inclusion as well as at 12 and 24 months to assess mood – depressive symptoms. The BDI has been recommended as a screening measure for depression in people with MS [18] and consists of 21 symptoms related to depression, 20 of which are self-rated from 0 (absent) to 3 (severe), and then one item from 0 (absent) to 2 (severe). The ratings for each item make up the total score, ranging from 0 to 62. The cut-off was a score of equal to or greater than 13 for depressive symptoms, which is in line with current recommendations [18]. The people with MS were categorized into those with Depressive symptoms (BDI score ≥ 13 at one or more points of data collection), and those with No depressive symptoms (BDI score < 13) throughout the study. The present study is an analysis based on the 173 people with MS who completed the BDI on all three occasions of data collection in the 2-year study and were residents within Stockholm County. A total of 27 people with MS who lacked a BDI score at one or more points of data collection (n = 15), or were residents outside of Stockholm County (n = 12), were not included.

Data on the use of inpatient (days) and outpatient (contacts) healthcare services were obtained from the computerised register of the Stockholm County Council. This register contains information regarding all healthcare contacts with care providers organized by the Stockholm County Council that have rendered notes (documentation of care) in the medical records. The types of outpatient contacts recorded in the register include visits, telephone consultations, and home visits. Searches were carried out for the whole study period, beginning six months before inclusion, and lasting until the data collection ended at 24 months, in total a period of 30 months.

Total outpatient care included both hospital outpatient care and primary care. Concerning outpatient neurology care and outpatient primary care, data were analyzed according to type of healthcare profession: physician; nurse; nurse aid; welfare officer; psychologist; occupational therapist; physiotherapist; dietician; and speech and language therapist. Data regarding the use of other healthcare services during the previous six months such as home-help services; salaried personal assistants; unsalaried informal care from partners and others; transportation service for the disabled; and periods of intense rehabilitation services at rehabilitation units organised by the private sector, were collected by interview according to a protocol at each point of data collection, thus covering a period of 30 months. A similar methodology has previously been used [1,2].

### Statistical analysis

Descriptive statistics were used to present demographic- and disease-related data and use of healthcare services in the total sample and in subgroups with regard to disease severity. The use of healthcare services was analysed with regard to disease severity, and categorized as EDSS mild, EDSS moderate, and EDSS severe. The Mann Whitney-*U* test was used for analyses of differences between people with MS with depressive symptoms versus no depressive symptoms in their use of healthcare services. For analyses of differences in other health services with regard to disease severity, the chi-square test was used. P-values less than or equal to 0.05 were considered statistically significant.

## Results

Demographic- and disease-related data at inclusion are presented in Table 1. Depressive symptoms were found in 71 (41%) people with MS at one or more points of data collection, whereas 102 (59%) had no depressive symptoms throughout the two-year study. Among the 111 people with MS and EDSS mild, 39 (35%) had depressive symptoms, whereas 72 (65%) had no depressive symptoms. Thirty people with MS had EDSS moderate, 16 (53%) with, and 14 (47%) without depressive symptoms; of the 32 that had EDSS severe, there were 16 (50%) with, and 16 (50%) without depressive symptoms. The use of antidepressant drugs in people with depressive symptoms varied from 28% among those with EDSS mild, to 38% among those with EDSS moderate (Table 1, footnote a-c).

**Table 1:** Demographic- and disease-related data at inclusion for the total sample and with respect to presence or absence of depressive symptoms and with regard to disease severity

	**Total**	***EDSS mild***		***EDSS moderate***		***EDSS severe***	
		**Depressive symptoms**	**No depressive symptoms**	**Depressive symptoms**	**No depressive symptoms**	**Depressive symptoms**	**No depressive symptoms**
	**n = 173**	**n = 39**^**a**^	**n = 72**	**n = 16**^**b**^	**n = 14**	**n = 16**^**c**^	**n = 16**
Women, n (%)	116 (67)	28 (72)	51 (71)	9 (56)	7 (47)	10 (62)	11 (69)
Mean age, years (sd)	46 (12)	42 (13)	43 (11)	47 (9)	55 (10)	56 (11)	53 (12)
Cohabiting, n (%)	123 (71)	31 (79)	51 (71)	11 (69)	9 (64)	11 (69)	11 (69)
Working full- or part-time, n (%)^d^	114 (66)	24 (65)	59 (84)	7 (47)	7 (64)	0	7 (46)
Immunomodulatory treatment, n (%)	146 (84)	30 (77)	57 (79)	15 (94)	14 (100)	14 (88)	16 (100)
Mean years since diagnosis, (sd)	13 (10)	9 (7)	12 (10)	15 (10)	15 (10)	19 (9)	20 (11)
Disease severity, n (%)							
EDSS mild 1.0-3.5	111 (64)	1.8 (1.0-3.5)	1.8 (1.0-3.5)				
EDSS moderate 4.0-5.5	30 (17)			4.7 (4.0-5.5)	4.6 (4.0-5.5)		
EDSS severe 6.0-9.5	32 (18)					7.3 (6.0-8.0)	7.0 (6.0-8.0)
Disease course, n (%)							
Relapsing remitting	106 (61)	35 (90)	62 (86)	3 (19)	4 (29)	0	2 (13)
Secondary progressive	58 (34)	1 (2)	7 (10)	12 (75)	9 (64)	16 (100)	13 (81)
Primary progressive	9 (5)	3 (8)	3 (4)	1 (6)	1 (7)	0	1 (6)

^a^11 (28%) people with MS used antidepressant drugs.

^b^6 (38) people with MS used antidepressant drugs.

^c^5 (31%) people with MS used antidepressant drugs.

^d^based on PwMS < 65 years, n = 159.

### Hospital care (outpatient and inpatient) and primary care

All people with MS involved in the study had been in contact with hospital outpatient care. Among people with depressive symptoms, a larger proportion, 96%, had contacts within primary care, compared to 84% among those with no depressive symptoms (p = 0.006). Furthermore, among people with depressive symptoms, a larger proportion, 32%, had received inpatient care, compared to 16% among those with no depressive symptoms (p = 0.01).

Among people with EDSS mild, larger proportions of those with depressive symptoms compared to those with no depressive symptoms were found receiving care from the following areas: nurses in hospital outpatient care (85% vs 61%, p = 0.01); speech/language therapists in hospital outpatient care (8% vs 0%, p = 0.04); psychiatric departments (20% vs 0%, p < 0.001); other departments (64% vs 43%, p = 0.03); total primary care (95% vs 79%, p = 0.02); and inpatient care (28% vs 12%, p = 0.02). Table 2 presents the number of users of healthcare services, the number of outpatient contacts, and the days of inpatient care, all categorized with respect to the presence or absence of depressive symptoms, and with regard to disease severity. Among people with EDSS mild, those with depressive symptoms were found to have a larger number of healthcare contacts with regard to total hospital outpatient care and to have utilised the following healthcare professionals: neurologists; nurses; welfare officers; speech/language therapists; psychiatric departments; and other departments. There was no difference regarding the number of hospital outpatient contacts with psychologists. With regard to total primary care, there was a larger number of healthcare contacts among people with depressive symptoms. No people with MS and EDSS mild had been in contact with psychologists within primary care. There was also a tendency to have a larger number of contacts with welfare officers within primary care among people with depressive symptoms. The number of days in inpatient care was larger among those with depressive symptoms.

**Table 2:** Use of healthcare services categorized with respect to presence or absence of depressive symptoms and with regard to disease severity; number of users, number of outpatient contacts and days of inpatient care during 30 months

	***EDSS mild *(Number of users), mean/median/range**			***EDSS moderate *(Number of users), mean/median/range**			***EDSS severe *(Number of users), mean/median/range**		
	**Depressive symptoms**	**No depressive symptoms**	**p value**	**Depressive symptoms**	**No depressive symptoms**	**p value**	**Depressive symptoms**	**No depressive symptoms**	**p value**
	**n = 39**	**n = 72**		**n = 16**	**n = 14**		**n = 16**	**n = 16**	
Hospital outpatient care, total	(39)	(72)		(16)	(14)		(16)	(16)	
	26/17/9-78	18.0/13/5-150	**0.002**	32.4/21/12-100	28.6/16/7-108	0.20	28.5/26/8-62	28.5/23/6-6	0.88
Neurologist	(39)	(72)		(16)	(14)		(16)	(16)	
	10,6/9/5-35	8.5/8/5-16	**0.04**	11.7/11/4-23	10.9/11/6-16	0.33	10.8/10/8-19	12.3/12/6-22	0.75
Nurse	(33)	(44)		(13)	(10)		(11)	(10)	
	3.2/2/0-14	1.7/1/0-10	**0.002**	2.7/3/0-7	2.2/2/0-9	0.22	1.8/2/0-7	1.4/1/0-7	0.47
Welfare officer	(14)	(15)		(7)	(4)		(1)	(4)	
	3.8/0/0-33	0.7/0/0-13	**0.03**	5.8/0/40	2.4/0/0-12	0.43	0.1/0/0-1	0.8/0/0-6	0.14
Physiotherapist	(8)	(10)		(10)	(3)		(4)	(6)	
	1.9/0/0-35	2.7/0/0-129	0.39	5.9/0/0-53	11.2/0/0-83	0.15	5.0/0/0-46	7.3/0/0-36	0.45
Occupational therapist	(6)	(8)		(3)	(1)		(3)	(3)	
	0.4/0/0-5	0.4/0/0-9	0.55	0.8/0/0-11	0.1/0/0-2	0.40	0.2/0/0-2	1.0/0/0-11	0.91
Dietician	(3)	(4)		(4)	(1)		(2)	(5)	
	0.1/0/0-2	0.1/0/0-4	0.70	0.3/0/0-2	0.1/0/0-1	0.19	0.2/0/0-2	0.3/0/0-1	0.27
Speech/language therapist	(3)	0		(2)	(2)		(2)	(4)	
	0.0/0/0-8		**0.02**	1.4/0/0-18	0.1/0/0-1	1.0	0.8/0/0-11	0.6/0/0-6	0.42
Psychologist	(3)	(9)		(1)	(1)		(4)	0	
	0.3/0/0-6	0.9/0/0-42	0.47	0.1/0/0-1	0.2/0/0-3	0.10	1.3/0/0-7		**0.04**
Emergency Room	(13)	(22)		(6)	(4)		(5)	(6)	
	0.7/0/0-8	0.4/0/0-4	0.62	0.6/0/0-3	0.2/0/0-1	0.44	0.5/0/0-4	0.6/0/0-3	0.64
Ophthalmology	(10)	(15)		(5)	(2)		(2)	(4)	
	0.6/0/0-6	0.6/0/0-19	0.56	0.6/0/0-6	0.3/0/0-4	0.32	0.1/0/0-1	0.3/0/0-2	0.36
Urology	(3)	(5)		(1)	(2)		(6)	(5)	
	0.3/0/0-13	0.2/0/0-7	0.91	0.1/0/0-1	0.5/0/0-4	0.76	1.6/0/0-10	1.1/0/0-6	0.69
Psychiatry	(8)	0		(3)	(1)		0	0	
	2.6/0/0-64		**<0.001**	1.9/0/0-27	0.1/0/0-1	0.34			
Other^a^	(25)	(31)		(12)	(7)		(11)	(9)	
	49.0/0/0-33	2.1/0/0-47	**0.01**	6.0/1/0-38	2.4/0/0-15	0.62	5.9/2/0-25	3.3/2/0-18	0.47
Primary care, total	(37)	(57)		(15)	(13)		(16)	(16)	
	14.4/4/0-104	8.5/3/0-80	**0.05**	45.2/15/0-191	46.7/16/0-245	0.85	116.6/94/4-482	87.4/26/4-733	**0.02**
Physician	(33)	(51)		(14)	(12)		(9)	(16)	
	3.5/2/0-12	3.3/2/0-31	0.24	3.3/3/0-17	3.3/2/0-10	0.80	6.1/4/0-21	3.4/2/1-7	0.85
Nurse	(18)	(39)		(12)	(8)		(16)	(15)	
	6.4/1/0-115	3.6/1/0-45	0.68	22.8/3/0-121	37.4/2/0-241	0.69	63.5/36/1-315	54.8/6/0-657	**0.05**
Occupational therapist	(6)	(4)		(9)	(4)		(13)	(11)	
	0.4/0/0-7	0.2/0/0-7	0.08	3.0/2/0-14	1.5/0/0-9	0.20	10.2/7/0-61	5.1/3/0-19	0.27
Physiotherapist	(7)	(5)		(6)	(3)		(11)	(9)	
	3.7/0/0-98	0.8/0/0-42	0.07	8.6/0/0-94	4.3/0/0-28	0.46	30.0/11/0-147	15.0/4/0-56	0.44
Nurse aid	(3)	(7)		(5)	(1)		(10)	(5)	
	0.2/0/0-5	0.5/0/0-30	0.76	4.9/0/0-57	0.1/0/0-1	0.09	16.8/3/0-121	8.2/0/0-107	0.10
Psychologist	0	0		(2)	0		(2)	(1)	
				1.8/0/0-19		0.19	0.2/0/0-3	0.6/0/0-11	0.63
Dietician	0	0		0	0		(1) 0.1/0/0-2	0	0.35
Welfare officer	(2)	0		0	(1)		0	0	
	0.1/0/0-3		0.06		3.0/0/0-42	0.32			
Hospital inpatient care^b,c^	(11)	(9)		(5)	(1)		(7)	(6)	
	3.9/0/0-75	0.5/0/0-8	**0.04**	4.4/0/0-29	1.0/0/0-14	0.28	9.3/0/0-79	2.2/0/0-13	0.47

^a^Dermatology, endocrinology, gynecology, hematology, medicine, oncology, surgery.

^b^Gynecology, neurology, medicine, infection, orthopedics, psychiatry, surgery.

^c^Days.

Among people with EDSS moderate, no differences were found in proportions of users, number of contacts within hospital outpatient care, primary care or in number of days spent in inpatient care between people with, versus without, depressive symptoms.

Among people with EDSS severe and with depressive symptoms, the few areas of difference were a larger number of contacts with hospital outpatient psychologists, the total use of primary care, and the use of nurses within primary care. There were no differences regarding the number of hospital outpatient contacts with welfare officers or psychologists within primary care. No people with EDSS severe had been in contact with psychiatric departments or with welfare officers within primary care.

### Other health services

Use of other health services in people with MS, categorized with respect to presence or absence of depressive symptoms and with regard to disease severity, is presented in Table 3. Among people with EDSS mild, larger proportions of those with depressive symptoms received informal care from partners and others, and larger proportions had undergone intense rehabilitation periods compared to people with no depressive symptoms. Among people with EDSS moderate and EDSS severe, no differences were found.

**Table 3:** Use of salaried services, informal care and of other types of services in people with MS, categorized with respect to presence or absence of depressive symptoms and with regard to disease severity; number of people receiving the services during 30 months

**Type of service, users (%)**	***EDSS mild***			***EDSS moderate***			***EDSS severe***		
	**Depressive symptoms n = 39**	**No depressive symptoms n = 72**	**p value**	**Depressive symptoms n = 16**	**No depressive symptoms n = 14**	**p value**	**Depressive symptoms n = 16**	**No depressive symptoms n = 16**	**p value**
Personal assistance	2 (5)	0	0.12	2 (12)	0	0.48	6 (38)	7 (44)	0.72
Home help service	1 (3)	0	0.35	2 (12)	4 (29)	0.38	8 (50)	6 (38)	0.48
Informal care	35 (90)	6 (8)	**0.02**	14 (88)	13 (93)	0.62	16 (100)	14 (88)	0.48
Intense rehabilitation period	11 (28)	8 (11)	**0.02**	13 (81)	8 (57)	0.15	12 (75)	13 (81)	0.67
Transportation service for the disabled	8 (20)	6 (8)	0.06	15 (94)	10 (71)	0.10	16 (100)	16 (100)	1.00

## Discussion

This study explored the use of health services in people with MS, with and without depressive symptoms during a period of 30 months, with regard to disease severity. The results showed that people with EDSS mild and depressive symptoms used more hospital outpatient care and primary care, and spent more days in inpatient care compared to people with EDSS mild and no depressive symptoms. Furthermore, the results showed that those people with EDSS mild and depressive symptoms received more informal care and intense rehabilitation periods than did people with EDSS mild with no depressive symptoms.

The larger amount of contacts with welfare officers in hospital outpatient care and contacts with psychiatric departments among people with EDSS mild and depressive symptoms is not surprising, and may in fact reflect an adequate supply of healthcare in line with current recommendations of treatment for depression in people with MS [18]. On the other hand, no clear pattern of healthcare use with regard to specific services was found, but instead, there was a noticeable general increase involving a number of different services. Similar results were found in a previous study of healthcare use among people with MS and fatigue based on the same cohort of people [2]. However, in the present study, the types of services where an increased use were found differ from the previous study; two examples of this would be the larger number of contacts with nurses in outpatient hospital care, and more total days spent in inpatient care. There are several possible explanations for these results. It has been shown that patients rate the impact of mental health as more important than their physicians do [25], and that people with disability differ in their perception of rehabilitation needs compared with their nominated key professionals [26]. Furthermore, depressive symptoms among people with MS have been found to be undertreated [27], and only a minority of those with depressive symptoms in the present study used antidepressant drugs. Thus, the increased use of health services may reflect that depressive symptoms were not noted or not adequately treated, therefore leading to repeated contacts with the healthcare system. Other possible explanations for the results could be that people with MS and depressive symptoms perceive their disability as being greater than their physicians' perception [28]. Another possibility is that depressive symptoms are associated with secondary health conditions and so an increased use of healthcare follows, as shown in a previous study [3]. Since people with MS and depressive symptoms have been shown to use less-effective coping strategies [13], it is also plausible that they are more dependent on the healthcare system in the absence of self-management strategies.

No differences in the use of health services were found among people with MS and EDSS moderate, while those with EDSS severe coupled with depressive symptoms had more contacts with several healthcare professionals compared to those with EDSS severe and no depressive symptoms. However, the differences in use of health services among people with EDSS severe were not as evident compared with people with EDSS mild. The differences in the use of health services with regard to disease severity may partly be attributed to the fact that the EDSS mild group was the largest, while the other groups of disease severity may have lacked statistical power to detect differences. Another plausible explanation might be that among people with EDSS moderate and EDSS severe, there are many other disabilities besides depressive symptoms that contribute to an increased need for use of health services.

Psychiatric symptoms, such as depressive symptoms, for example, in people with MS are associated with high levels of distress among caregivers [29]. The finding in the present study that those with depressive symptoms also received more unsalaried informal care from partners and others adds further insights of the caregiver burden among caregivers to people with both MS and depressive symptoms. Caregiver burden is a very important issue with MS, since it is generally diagnosed in young adults, and many years of future caregiving are often involved. Recognition of caregiver burden is important in determining appropriate interventions for the families of people with MS [30].

The major strengths of the present study were the longitudinal design covering 30 months; the use of the BDI, a recommended screening measure for depressive symptoms in people with MS [18]; and the reliable data on healthcare use derived from the computerised register at the Stockholm County Council. However, data on the use of other healthcare services were collected by interview, and may thus be biased by the difficulties to recall information among the people with MS. Further, the groups of people with EDSS moderate and EDSS severe were quite small and may have lacked statistical power to detect differences in the use of healthcare services. The results should also be interpreted bearing in mind that the study was carried out within the Swedish healthcare system and differences in organisation and policies need to be considered before the results can be generalized to other healthcare systems.

## Conclusions

In conclusion, this study revealed a higher use of health services among people with both MS and depressive symptoms in comparison with those without depressive symptoms, especially among people with EDSS mild. The issues underlying these differences need to be explored further, as well as do the plausible implications for the organization of health services for people with MS and depressive symptoms. Furthermore, the life situations of caregivers of people with EDSS mild and depressive symptoms should be considered, and appropriate interventions supplied in order to diminish caregiver burden.

## Competing interests

Data collection was supported by an unrestricted grant from Biogen Idec. The authors declare that they have no competing interests.

## Authors’ contributions

CY participated in the design of the study, performed the statistical analyses and drafted the manuscript. SL performed the statistical analyses and helped to draft the manuscript. SJ participated in the design of the study and helped to draft the manuscript. All authors read and approved the final manuscript.

## Pre-publication history

The pre-publication history for this paper can be accessed here:

http://www.biomedcentral.com/1472-6963/13/365/prepub
